# MRSA Variant in Companion Animals

**DOI:** 10.3201/eid1812.120238

**Published:** 2012-12

**Authors:** Birgit Walther, Lothar H. Wieler, Szilvia Vincze, Esther-Maria Antão, Anja Brandenburg, Ivonne Stamm, Peter A. Kopp, Barbara Kohn, Torsten Semmler, Antina Lübke-Becker

**Affiliations:** Author affiliations: Freie Universität Berlin, Berlin, Germany (B. Walther, L. H. Wieler, S. Vincze, E.-M. Antão, B. Kohn, T. Semmler, A. Lübke-Becker);; Vet Med Labor GmbH, Ludwigsburg, Germany (A. Brandenburg, I. Stamm, P.A. Kopp)

**Keywords:** MRSA, companion animals, mecA, ST599, CC130, ST130, zoonotic novel mecA, bacteria, Staphylococcus aureus

## Abstract

Methicillin-resistant *Staphylocoocus aureus* (MRSA) harboring *mec*A_LGA251_ has been isolated from humans and ruminants. Database screening identified this MRSA variant in cats, dogs, and a guinea pig in Germany during 2008–2011. The novel MRSA variant is not restricted to ruminants or humans, and contact with companion animals might pose a zoonotic risk.

Worldwide, methicillin-resistant *Staphylococcus aureus* (MRSA) is a leading cause of infectious diseases in humans and animals (*1*). The staphylococcal cassette chromosome *mec* (SCC*mec*) harbors the *mec*A gene, which encodes an additional penicillin-binding protein 2a. In the presence of β-lactam antimicrobial drugs, this transpeptidase substitutes an essential cross-linking step in the process of cell-wall building (*2*). Eleven distinct SCC*mec* elements have been described (*3*). Recent reports of MRSA carrying a novel *mec*A homologue (*mec*A_LGA251_) of a predicted amino acid identity of 62% with other *mec*A allotypes raised awareness about these pathogens, which possibly remain undetected by conventional PCR approaches (*3*–*5*). This lack of detection might have led to underestimation of the novel MRSA variant among clinical samples of human and animal origin.

High-level congruence between *S. aureus* of animal and human lineages has been demonstrated (*6*), and nearly every sequence type (ST) reported for MRSA associated with infections in companion animals was also commonly found in humans (*1*). Because previous reports indicated that MRSA harboring *mec*A_LGA251_ originated from either human or ruminant hosts (*3*–*5*), we searched our database for companion animal isolates that displayed a MRSA phenotype but had failed to give a positive PCR result for *mec*A.

## The Study

From November 2008 through December 2011, MRSA of companion animal origin was routinely isolated from specimens submitted for diagnostic purposes to Vet Med Labor GmbH in Ludwigsburg, Germany, or to the Institute of Microbiology and Epizootics, Freie Universität Berlin, in Berlin, Germany. *S. aureus* was confirmed as described (*7*) and stored in glycerol stocks at −80°C.

PCR routinely used to confirm methicillin resistance and species identity had failed to produce a positive signal for *mec*A in 10 MRSA isolates from companion animals (2 isolates from dogs, 7 from cats, and 1 from a guinea pig) (*8*). We screened these 10 isolates for the *mec*A homologue by using the PCR method published by Cuny et al. (*5*) and sent the amplicons obtained to LGC Genomics GmbH (Berlin, Germany) for sequencing. Automated antimicrobial drug susceptibility testing was performed by using the bioMérieux VITEK 2 system (Nürtingen, Germany) according to the manufacturer’s instructions. The following drugs were tested according to Clinical and Laboratory Standards Institute guidelines M31–A3: penicillin, ampicillin–sulbactam, oxacillin, gentamicin, kanamycin, enrofloxacin, marbofloxacin, erythromycin, clindamycin, tetracycline, nitrofurantoin, chloramphenicol, and trimethoprim–sulfamethoxazole, (*9*). All isolates were further characterized by *spa* typing, multilocus sequence typing, and microarray hybridization by using the Alere Identibac *S. aureus* Genotyping chip (Alere Technologies GmbH, Jena, Germany) as described (*10*–*12*).

The presence of the *mec*A homologue was verified for all 10 isolates. All PCR amplicons demonstrated 100% identity with the DNA sequence of *mec*A_LGA251_ (National Center for Biotechnology Information no. FR821779.1). The strains originated from geographically diverse areas (5 federal states of Germany) and were isolated from different infection sites (Table). All strains were identified as MRSA by the VITEK 2 system (growth in the presence of 6 μg/mL cefoxitin according to the VITEK 2 Advanced Expert System), although oxacillin MICs were rather low (0.5 μg/mL) or moderately high (≥4 μg/mL) (Table). Phenotypic resistance toward non–β-lactams was not detected.

**Table:** Characteristics of 10 methicillin-resistant *Staphylococcus aureus* isolates harboring *mec*A_LGA251_ obtained from companion animals, Germany, 2008–2011*

IMT no.	Original no.	Year isolated	Host	Site	Clinical signs	Free-ranging animal	Federal state	OXA MIC†	*spa* type‡	ST§
17403	VB 999987	2008	Cat	Eye	Purulent infection	Yes	Rhineland-Palatinate	≥4	t10033	1945
21135	VB 964992	2010	Cat	Wound	Lymphadenitis	Yes	Bavaria	≥4	t843	130
21231	VB 971931	2010	Cat	Skin	Dermatitis	Unknown	Hessia	1	t1773	130
24068	VB 961584	2010	Cat	Tachea	Stridor	Yes	Hessia	0.5	t10006	599
25044	VB 969929	2010	Dog	Abscess	Tumor, dolor	No	Bavaria	2	t1694	599
25147	VB 969572–2	2010	Cat	Wound	Suture dehiscence	Yes	North Rhine-Westphalia	2	t278	599
25470	VB 972406	2010	Dog	Eye	Purulent infection	No	Bavaria	2	t1694	599
25715	VB 969935	2010	Guinea pig	Fistula	Purulent infection	No	Hessia	2	t843	130
28299	VB 952042	2011	Cat	Phlegmon	Dermatitis	Yes	Bavaria	1	t278	599
28429	IMT 2272/11	2011	Cat	Abscess	Fever	Yes	Berlin	≥4	t10009	130

*IMT, Institute of Microbiology and Epizootics, Freie Universität Berlin, Berlin, Germany; OXA, oxacillin; ST, sequence type; VB, Vet Med Labor GmbH, Ludwigsburg, Germany. All isolates were positive for *nuc* and negative for *mecA* according to PCR to detect MRSA (*8*), and all were positive for *mecA*_LGA251_ according to PCR to detect the novel *mec*A homologue (*5*). †Detected by the VITEK 2 system (bioMérieux, Nürtingen, Germany). ‡*Spa* repeats and *spa* type determined according to Harmsen et al. (*11*) and the Ridom SpaServer (http://spaserver.ridom.de). §Determined according to Enright et al. (*10*).

As has been described for atypical MRSA, 4 strains belonged to ST130 and 1 strain belonged to ST1945 (differs from ST130 by 1 allele) (*3*–*5*). The remaining 5 isolates were assigned to ST599 (differs from ST121 by 2 alleles) (Table). ST599 has been reported for methicillin-susceptible isolates from humans in Europe, Asia, and Africa (www.mlst.net). The Figure shows a minimum spanning tree based on 4,197 entries of the *S. aureus* multilocus sequence type database (http://saureus.mlst.net/) as of January 19, 2012 (Figure, panel A) and a detailed view of the branches and STs harboring strains with the novel *mec*A homologue published (Figure, panel B) (*3*–*5*).

**Figure:**
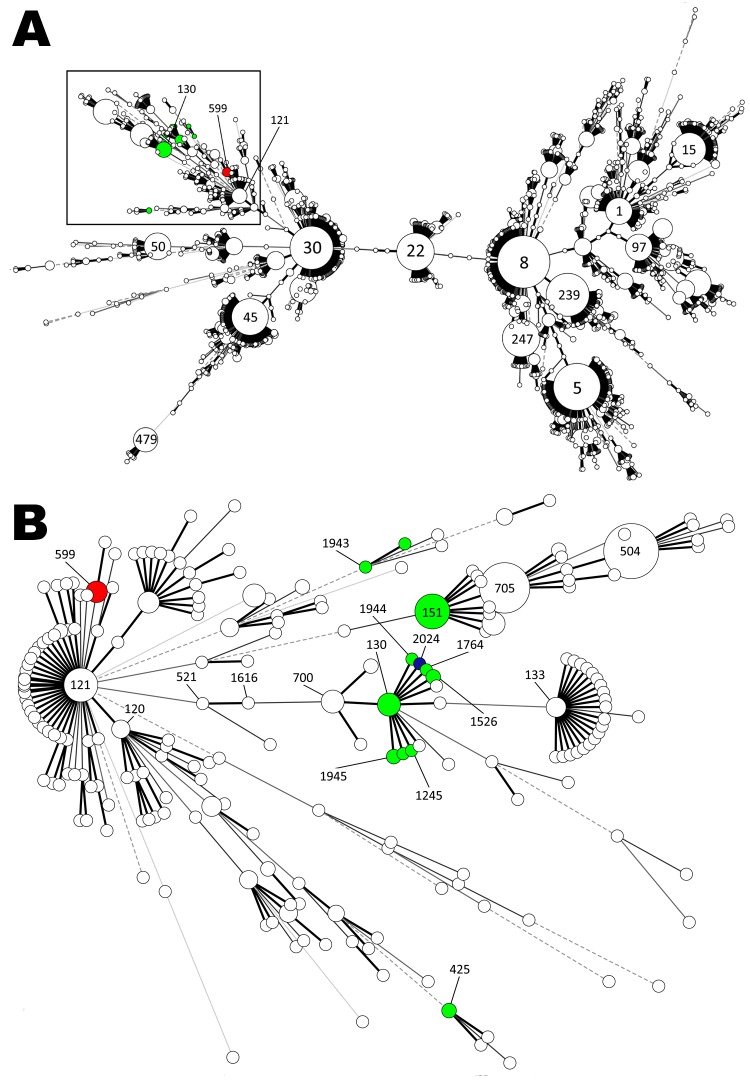
Minimum spanning tree based on multilocus sequence typing data from 4,197 *Staphylococcus aureus* strains (A) and an enlarged view of 1 phylogenetic group (B). Each circle represents a distinct sequence type (ST), and circle size is proportional to ST frequency. Green indicates *mec*A_LGA251_-positive *S. aureus* strains of companion animal origin reported in this study and sequence data from published multilocus sequence typing results (*3**–**5*); red indicates ST599 methicillin-resistant *S. aureus*; and blue represents ST2024 methicillin-sensitive *S. aureus* isolated from a wild rat.

Microarray hybridization data revealed that the *agr* type I and capsule type 5 seem to be associated with ST599 and *agr*III and that clonal complex (CC) 130 isolates harbor the capsule type 8 encoding gene. CC130 and ST599 isolates were positive for the surface-associated proteins *clf*A, *clf*B, *fnb*A, and *bbp*. All ST599 strains produced a positive hybridization result for 1 of the gene variants encoding the toxin responsible for toxic shock syndrome (*tst1* or *tst-bov*), and all but 1 of them harbored the enterotoxins C (*sec*) and L (*sel*), indicating the presence of an *S. aureus* pathogenicity island that encodes superantigens (*13*). Positive or ambiguous hybridization signals for *ccrB1*, *ccrA3*, and *ccrB3* were obtained for 5 isolates, suggesting the presence of the SCC*mec*XI in those strains, according to the results of Shore et al. (*4*).

## Conclusions

Our findings of CC130 and ST599 MRSA harboring *mec*A_LGA251_ in several companion animal species suggest that in Germany, the presence of the *mec*A homologue in MRSA is not exclusively associated with CC130. This finding supports the hypothesis that some, if not all, MRSA strains that harbor the novel *mec*A variant can cause infections among a broad variety of hosts, as has been shown for MRSA of human, equine, canine, and other companion animal origins (*1*,*7*). All currently known *mec*A_LGA251_–carrying MRSA were observed in a distinct section of the *S. aureus* population (Figure, panel B). Whether this phylogenetic group possesses the ability to integrate the novel *mec*A variant needs to be further investigated.

In the past, *mec*A_LGA251_–carrying MRSA could have been misidentified as methicillin sensitive by routine PCR. However, all isolates were correctly identified as MRSA by the VITEK 2 system, as reported (*4*).

Of the 10 isolates, 7 were found in specimens from cats. A recent study identified cats as a potential natural reservoir for *S. aureus* of CC133, a genetic lineage that has also been reported for *S. aureus* of ruminant origin (*14*). Moreover, we have identified a CC130 strain (MSSA_ST2024, t8403) from a wild rat (IMT21250; ID4035) (www.mlst.net). In addition, CC130 MRSA containing the *mec*A homologue has only recently been reported for isolates from humans in Germany (*5*).

Although many investigators focus on livestock-associated MRSA, and because particular companion animal lineages of MRSA seem to be lacking, transmission of MRSA between companion animals and human family members in close proximity might be underestimated, especially in cases of recurrent infection (*15*). The emergence of MRSA harboring the novel *mec*A homologue has consequences for the verification methods for MRSA used in veterinary medicine; implementation of new methods will be inevitable. Their supposed restriction to only a few genetic lineages and the potential risk for interspecies transmission of atypical MRSA between companion animals and their owners in household environments needs further elucidation.
